# Mortality prediction in patients with isolated moderate and severe traumatic brain injury using machine learning models

**DOI:** 10.1371/journal.pone.0207192

**Published:** 2018-11-09

**Authors:** Cheng-Shyuan Rau, Pao-Jen Kuo, Peng-Chen Chien, Chun-Ying Huang, Hsiao-Yun Hsieh, Ching-Hua Hsieh

**Affiliations:** 1 Department of Neurosurgery, Kaohsiung Chang Gung Memorial Hospital and Chang Gung University College of Medicine, Taiwan; 2 Department of Plastic Surgery, Kaohsiung Chang Gung Memorial Hospital and Chang Gung University College of Medicine, Taiwan; 3 Department of Trauma Surgery, Kaohsiung Chang Gung Memorial Hospital and Chang Gung University College of Medicine, Taiwan; National Yang-Ming University, TAIWAN

## Abstract

**Background:**

The purpose of this study was to build a model of machine learning (ML) for the prediction of mortality in patients with isolated moderate and severe traumatic brain injury (TBI).

**Methods:**

Hospitalized adult patients registered in the Trauma Registry System between January 2009 and December 2015 were enrolled in this study. Only patients with an Abbreviated Injury Scale (AIS) score ≥ 3 points related to head injuries were included in this study. A total of 1734 (1564 survival and 170 non-survival) and 325 (293 survival and 32 non-survival) patients were included in the training and test sets, respectively.

**Results:**

Using demographics and injury characteristics, as well as patient laboratory data, predictive tools (e.g., logistic regression [LR], support vector machine [SVM], decision tree [DT], naive Bayes [NB], and artificial neural networks [ANN]) were used to determine the mortality of individual patients. The predictive performance was evaluated by accuracy, sensitivity, and specificity, as well as by area under the curve (AUC) measures of receiver operator characteristic curves. In the training set, all five ML models had a specificity of more than 90% and all ML models (except the NB) achieved an accuracy of more than 90%. Among them, the ANN had the highest sensitivity (80.59%) in mortality prediction. Regarding performance, the ANN had the highest AUC (0.968), followed by the LR (0.942), SVM (0.935), NB (0.908), and DT (0.872). In the test set, the ANN had the highest sensitivity (84.38%) in mortality prediction, followed by the SVM (65.63%), LR (59.38%), NB (59.38%), and DT (43.75%).

**Conclusions:**

The ANN model provided the best prediction of mortality for patients with isolated moderate and severe TBI.

## Background

Identifying patients with traumatic brain injury (TBI) with high risk of mortality is important to maximize the resource for trauma care, and so that family members receive appropriate counsel and treatment decisions [1, 2]. One widely applied predictor of mortality outcome is the Trauma and Injury Severity Score (TRISS), which shows good discrimination in identifying the patients with TBI at high risk of mortality [3]. However, the model was not specifically designed for use in patients with TBI and is not always associated with high performance [4]. For patients with TBI, two prediction models (Corticosteroid Randomization after Significant Head Injury [CRASH] and the International Mission for Prognosis and Analysis of Clinical Trials in Traumatic Brain Injury [IMPACT]) based on large clinical trial datasets have shown good discrimination and have enabled accurate outcome predictions [5–7]. However, these two models lack the precision required for use on the individual patient level [8, 9].

Currently, machine learning (ML) has been successfully applied to aid in clinical diagnosis and prognosis prediction [10, 11]. In some field-specific datasets, many ML techniques have shown significantly better predictive power than more conventional alternatives [12]. Applied ML techniques in the clinical setting include, but are not limited to, LR, support vector machine (SVM), decision trees (DT), Bayes classification, and artificial neural networks (ANN). This study aimed to construct an optimal model of ML for mortality prediction among patients with moderate and severe TBI by using data from a population-based trauma registry in a level I trauma center.

## Methods

### Subject and data preparation

This study was approved by the Institutional Review Board (IRB) of Chang Gung Memorial Hospital with approval number 201700014B0. Informed consent was waived according to the regulation of the IRB. Detailed patient information between January 2009 and December 2015 was retrieved from the Trauma Registry System of the hospital. The adult patient cohort included those who were ≥ 20 years of age and hospitalized for the treatment of moderate and severe TBI, defined as an AIS score ≥ 3 points in the head (moderate TBI, AIS 3–4; severe TBI, AIS 5) [13, 14]. Polytrauma patients who had additional AIS scores ≥ 3 points corresponding to any other region of the body were excluded from this study [14]. Enrolled patients were divided into a training set (a 6-year span between 2009 and 2014) for generation of a plausible model under supervised classification, and a test set (a 1-year span in 2015) to test the performance of the model. Patients with missing data were not included in the dataset for analysis. The retrieved patient information included the following variables: age, sex, helmet-wearing status, pre-existed co-morbidities, such as coronary artery disease (CAD), congestive heart failure (CHF), cerebral vascular accident (CVA), diabetes mellitus (DM), end-stage renal disease (ESRD), and hypertension (HTN). Glasgow coma scale (GCS) score and vital signs, including temperature, systolic blood pressure (SBP), heart rate (HR), and respiratory rate (RR) were collected upon patient arrival at the emergency department. Blood-drawn laboratory data at the emergency room, including white blood cell count (WBC), red blood cell count (RBC), hemoglobin (Hb), hematocrit (Hct), platelets, blood urine nitrogen (BUN), creatinine (Cr), alanine aminotransferase (ALT), aspartate aminotransferase (AST), sodium, potassium, and glucose was also collected. TBI-related diagnoses, such as epidural hematoma (EDH), subdural hematoma (SDH), subarachnoid hemorrhage (SAH), and intracerebral hematoma (ICH) were assessed, as were ISS and the in-hospital mortality of patients during admission. The in-hospital mortality included those caused by the injury directly to the brain or by the associated complication such as pneumonia or sepsis. Finally, a total of 1734 (1564 survival and 170 non-survival) and 325 (293 survival and 32 non-survival) patients comprised the training and test sets, respectively.

### ML classifications

#### Logistic regression (LR)

In this study, the LR classifier used glm function in the stats package in R3.3.3 (R Foundation for Statistical Computing, Vienna, Austria). A stepwise LR analysis was used to control the effects of confounding variables to identify independent risk factors for mortality.

#### Support vector machine (SVM)

The SVM classifier used the tune.svm & svm function in the e1071 package in R with the radial basis function to handle non-linear interactions [15]. The optimal operating point was estimated using a grid search with a 10-fold cross-validation varying the penalty parameter C, which determined the tradeoff between fitting error minimization and model complexity, and hyper-parameter γ, which defined the nonlinear feature transformation onto a higher dimensional space and controlled the tradeoff between error due to bias and variance in the model [15].

#### Decision tree (DT)

In this study, the classification and regression trees (CART) of DT were used based on the Gini impurity index to achieve the best overall split [16], with the rpart function in the rpart package in R to enable better prediction through progressive binary splits in a combined approach for both nonparametric and nonlinear variables.

#### Naïve bayes (NB)

The NB classifier—deemed as the simplest of Bayes classifiers—makes the assumption that input variables are conditionally independent of each other given the classification [17, 18]. Despite its simplicity, its performance is comparable to conventional or more sophisticated methods [19, 20], and it has yielded good results in mortality-classification settings [21]. In this study, the NB classifier used the naiveBaye function in the e1071 package in R.

#### Artificial neural networks (ANN)

The ANN is constructed from a set of neurons that exchange signals with each other via an interconnected network. Each connection has a numeric weight that can be adjusted during training of the network, making the system adaptive to input patterns and capable of revealing previously unknown relationships between given input and output variables [22–24]. In this study, the ANN classifier used a feed-forward neural network with the nnet function in the nnet package in R. The tuning parameters included the number of nodes in the hidden layer optimized between 1 and 20. For the training process, maximal iterations and decay were selected as 1000 and 0.001, respectively. To avoid over-fitting, iterations occurred until the error did not significantly decrease.

### Performance of the ML classifiers

Model predictive performance regarding accuracy, sensitivity, specificity and the area under the curve (AUC) of the receiver operator characteristic curves (ROCs) corresponding to the two different models was measured. A nonparametric approach to the analysis of the AUC under correlated ROCs using the roc & roc.test function in the pROC package in R was pursued [25]. The predicted probabilities against binary events was validated using the val.prob function in the rms package in R. Somers’ Dxy measured probability of concordance minus the probability of discordance between predicted outcomes and observed outcomes and was used to assess the predictive discrimination [26]. The coefficient of determination, R^2^, is a statistic that will give some information about the goodness of fit of a model [27]. An R^2^ of 1 indicates that the regression line perfectly fits the data. The Brier score indicated an overall measure of model performance by defining the mean squared error between the predicted probabilities and the actual outcomes [28]. Brier scores vary between 0 and 1, a lower score indicating higher accuracy.

### Statistical analyses

All statistical analyses were performed using SPSS 20.0 (IBM Inc., Chicago, IL, USA) and R 3.3.3. For continuous variables, we used student t-tests to analyze normally distributed data, while Kolmogorov-Smirnov or Mann-Whitney U tests were used to compare non-normally distributed data. For categorical variables, we used Chi-square tests to determine the significance of the association between variables. Results are presented as mean ± standard deviation, and a p-value < 0.05 was considered statistically significant.

## Results

### Patient demographics and injury characteristics

**Fig 1:**
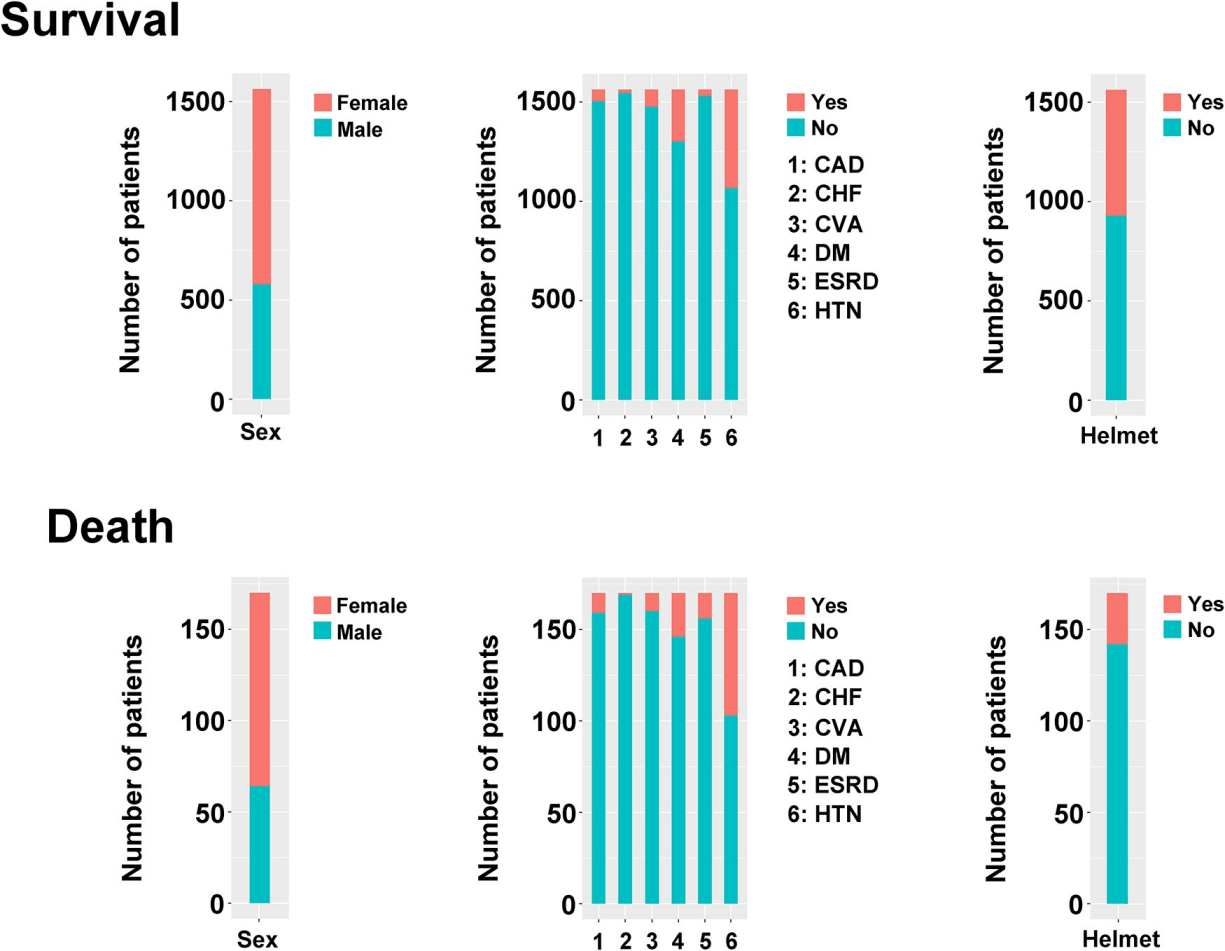
Patient demographics and injury characteristics.

As shown in Fig 1, there was no significant difference in sex, CAD, CHF, CVA, and DM between patients that survived TBI and those that did not. In contrast, ESRD and HTN incidences were higher in the fatality group than in the group of patients that survived. In addition, fewer patients in the fatality group had worn a helmet when compared to patients in the survival group. A statistically significant difference in age, ISS, GCS, glucose, temperature, HR, RBC, Hb, Hct, platelets, and K was also found between groups (S1 Fig). Because correlation coefficients between Hb and Hct, BUN and Cr, and AST and ALT were similar, only one of the three representative variables (i.e., Hct, BUN, and AST) was selected for further ML classification to prevent the inclusion of duplicate parameters. Therefore, 25 total variables were used for ML classifier imputation.

### Performance of ML classifiers in the training set

LR identified 12 predictors (age, helmet status, platelets, DM, SDH, Hct, temperature, ICH, GCS, glucose, ISS, and ESRD) as independent risk factors for mortality (S2 Fig).

The SVM classifier was performed for the prediction of mortality, taking input with all 25 variables with two parameters (C, γ) being determined by a grid search of 2^x^, where x is an integer between -20 and 4 for C and between -20 and -4 for γ. The values which gave the highest 10-fold cross-validation accuracy were C = 0.00003 and γ = 0.000977.

**Fig 2:**
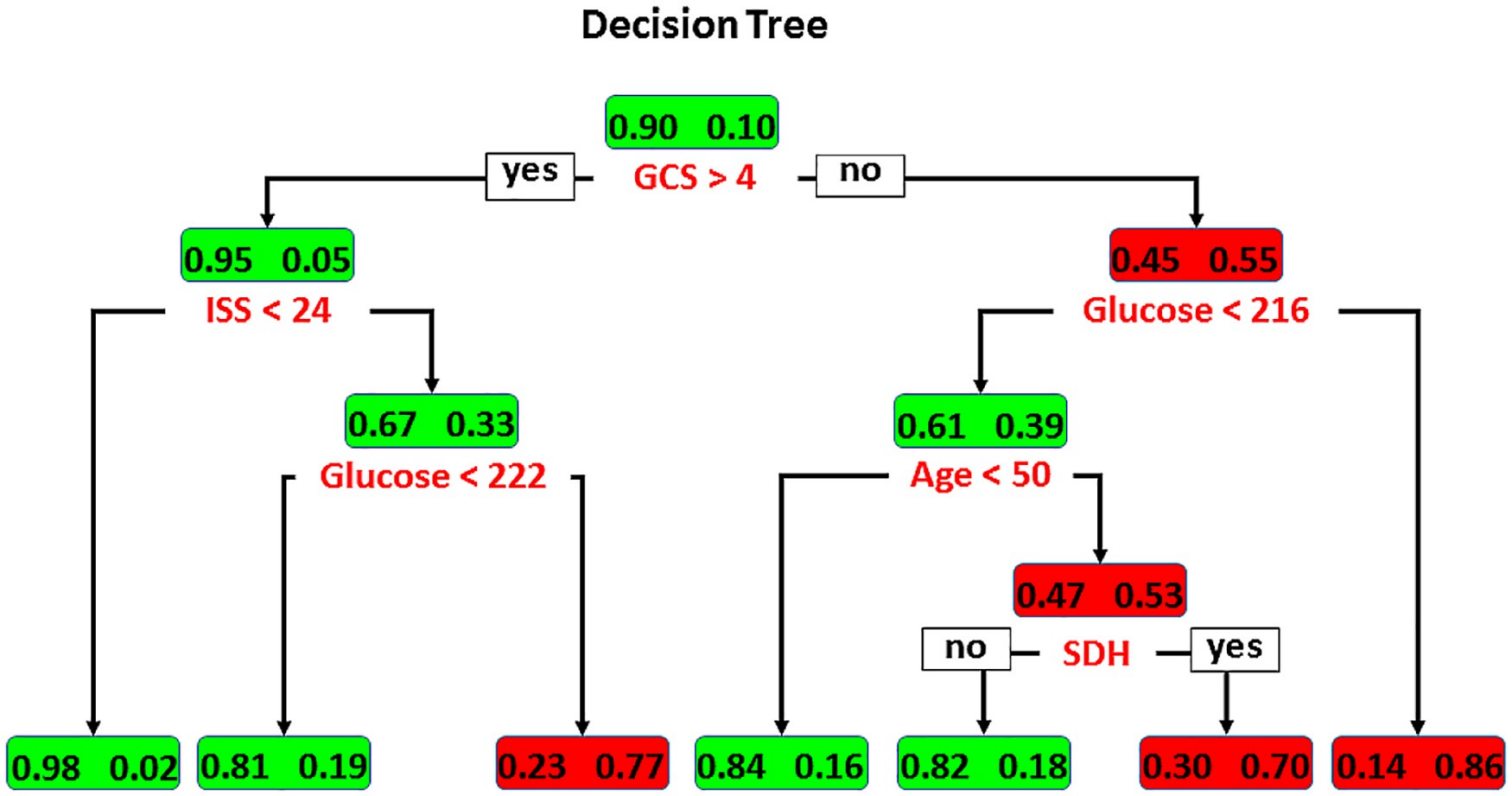
Illustration of the DT model for predicting mortality in patients with isolated moderate and severe TBI. Boxes denote the percentage of patients analyzed with discriminating variables; survivors and non-survivors are indicated by green and red colors, respectively.

In the DT model (Fig 2), the GCS was identified as the initial split variable, with an optimal cut-off value of > 4. Among patients having a GCS < 4, high glucose levels of ≥ 216 mg/dL indicated a fatal outcome. In patients with glucose levels < 216 mg/dL, the next best predictor of mortality was age, with an optimal cut-off < 50 years. In patients ≥ 50-years-old, existence of SDH presented as a predictor of fatal outcome. In addition, among patients with GCS > 4, an ISS ≥ 25 and glucose levels ≥ 222 mg/dL were selected as significant variables for the prediction of a fatal outcome.

**Fig 3:**
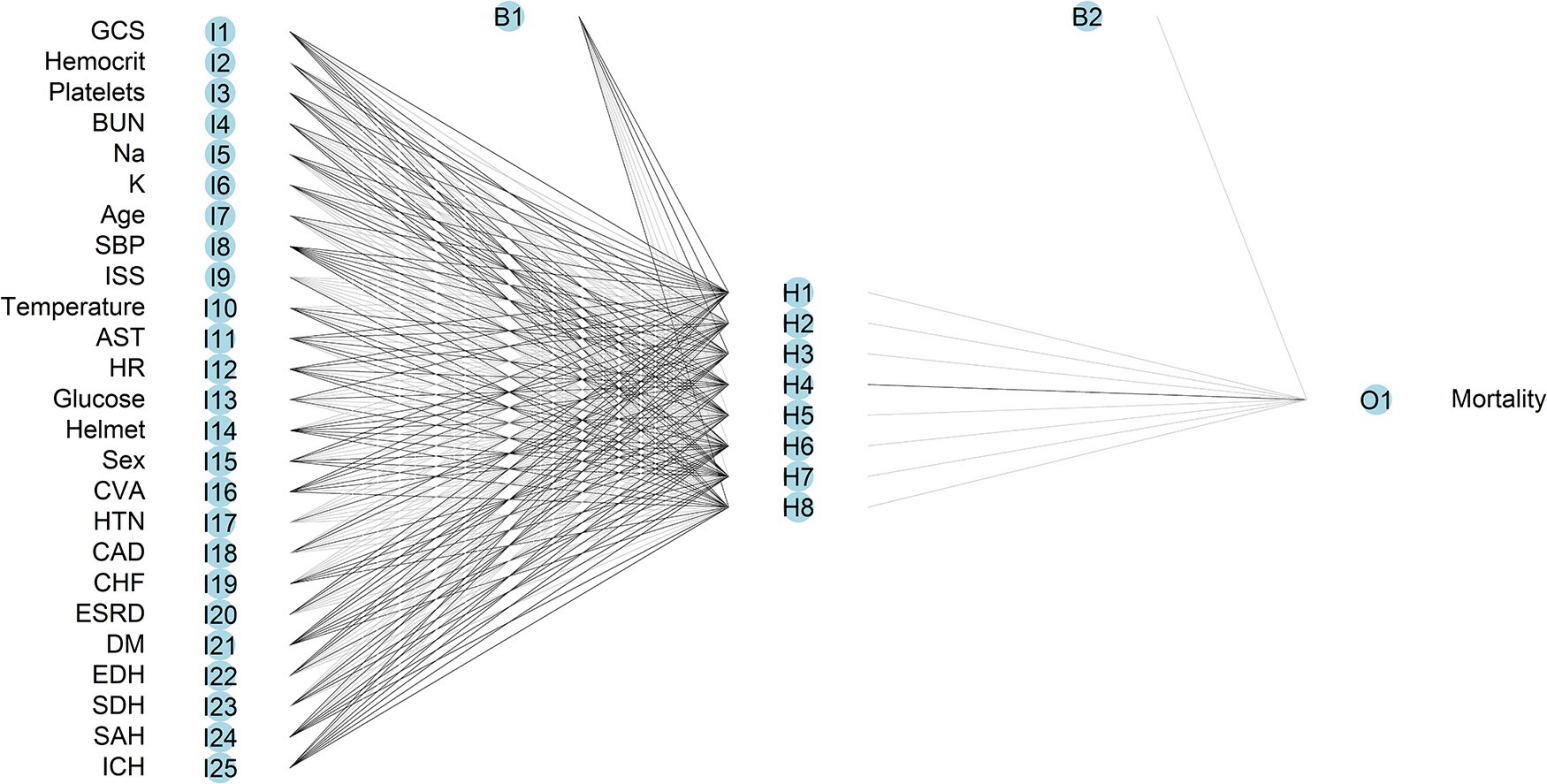
Architecture of the three-layered feed-forward ANN.

The constructed ANN model includes 25 inputs, one bias neuron in the input layer, eight hidden neurons, one bias neuron in the hidden layer, and one output neuron (Fig 3). A single output node indicated the probability of death.

**Fig 4:**
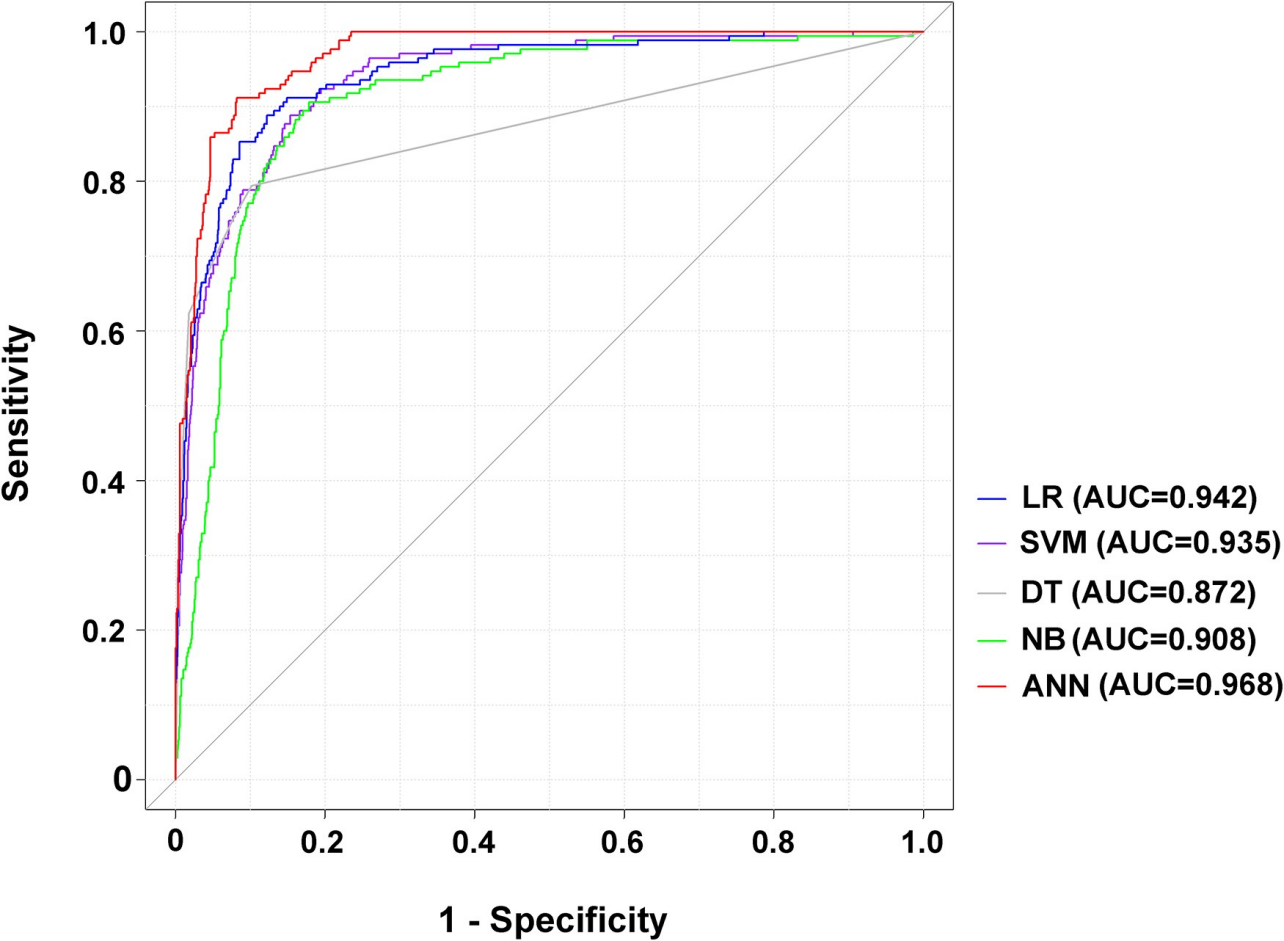
ROC curves for the LR, SVM, DT, NB, and ANN models in predicting the mortality of patients with isolated moderate and severe TBI.

**Fig 5:**
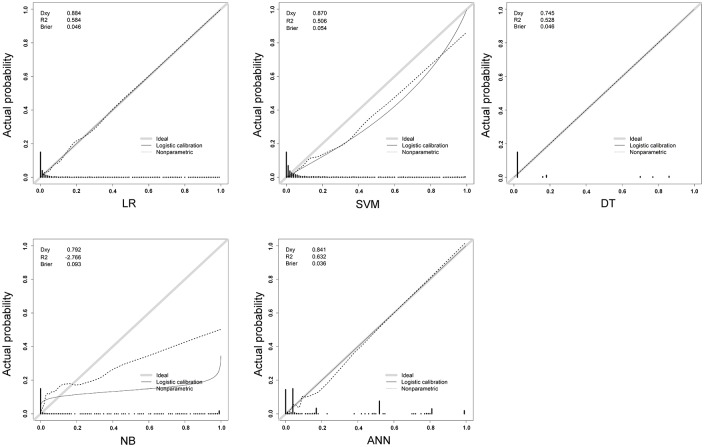
Calibration curves by the LR, SVM, DT, NB, and ANN models in predicting the mortality of patients with isolated moderate and severe TBI.

As shown in Table 1, all ML models, with the exception of the NB, achieved an accuracy of more than 90%. As the high survival among investigated patients rate likely accounts for the observed accuracy and specificity in mortality prediction, we further focused on the sensitivity of different ML models. In the established model, ANN had the highest sensitivity (80.59%), followed by the NB (73.53%), SVM (64.12%), DT (62.35%), and LR (53.89%). All five ML models had a specificity of more than 90%. In comparing AUCs of the ROCs among the five ML models for the training set (Fig 4), the ANN had a significantly higher AUC (0.968) than the other four ML models (S1 Table). Both LR (0.942) and SVM (0.935) had significantly higher AUCs than the NB (0.908) or DT (0.872); however, there was no significant difference in AUC between LR and the SVM. According to AUC comparisons, and in consideration of prediction sensitivity, the ANN was determined to be the best algorithm to predict mortality. The calibration curves of these five predictions demonstrated that LR, DT, and ANN plotted a nonparametric line close along the ideal diagonal line (Fig 5), while LR had the highest Dxy (0.884) and ANN had the highest R^2^ (0.632) and lowest Brier score (0.036).

**Table 1:** Mortality prediction performance (i.e., accuracy, sensitivity, and specificity) for the LR, SVM, DT, NB, and ANN models on training and test sets.

Methods	Train			Test		
	Accuracy	Sensitivity	Specificity	Accuracy	Sensitivity	Specificity
LR	93.66%	53.89%	98.08%	93.54%	59.38%	93.54%
SVM	92.96%	64.12%	96.10%	92.50%	65.63%	95.22%
DT	94.69%	62.35%	98.21%	92.92%	43.75%	98.29%
NB	89.56%	73.53%	91.30%	86.15%	59.38%	89.08%
ANN	93.94%	80.59%	95.40%	92.00%	84.38%	92.83%

LR, logistic regression; SVM, support vector machine; DT, decision trees; NB, Naive Bayes; and ANN, artificial neural networks.

### Performance of ML classifiers in the test set

For the test set, with the exception of the NB, all ML models achieved similar rates of accuracy (~92% to 93.5%) in terms of their ability to predict mortality (Table 1). In addition, these models achieved specificity rates that were greater than 92%. The ANN still displayed the highest sensitivity (84.38%) in its ability to predict mortality, followed by the SVM (65.63%), LR and NB (59.38%), and DT (43.75%).

## Discussion

In this study, both LR and the SVM had significantly higher AUCs than the NB and DT. It has been reported that the NB classifier operates under the assumption that independence is valid. When two variables are related, however, the NB may place too much weight on them and too little weight on other variables, resulting in classification bias [17, 18]. In addition, the DT may lead to overestimation of the importance of included risk factors or may exclude other potential confounding factors that could influence actual risk [29]. On the other hand, the SVM boundary is only minimally influenced by outliers [30] and the employment of kernels help the model learn non-linear decision boundaries, allowing the classifier to solve more complex data than linear analyses methods, such as the LR model [31]. However, this advantage could not be identified in the current study, as there was no significant difference in AUC values between the LR and SVM. The exceptional performance of the LR model implies that most patients that died following TBI could be explained by a relatively small set of independent predictors that fit the logistic model assumptions well, such as old age [32], hyperglycemia [33], GCS scores [32, 34], and the presence of SDH [35]. Therefore, to improve the predictive performance of models than the LR, additional data or different approaches may be considered for the prediction task.

One study that predicted 10 609 trauma patient outcomes, using 16 anatomic and physiologic predictor variables, revealed that the ANN exceeded the TRISS model in terms of its ability to predict mortality, with an AUC of 0.912 compared to 0.895 for the TRISS [36]. Furthermore, the ANN model has been shown to be more accurate and to have better overall performance than LR model in predicting in-hospital mortality for patients receiving mechanical ventilation [37] as well as for patients in critical care [38]. For patients with TBI, the CRASH prognostic model shows good discrimination for 14-day mortality prediction, with an AUC of 0.89, while the IMPACT prognostic model shows good discrimination for 6-month mortality predictions, with an of AUC 0.80 [3]. In this study, we demonstrated that the ANN had a significantly higher AUC than the other four ML models when predicting the mortality of patients with isolated moderate and severe TBI. Moreover, the ANN retained the highest sensitivity (84.38%) among the investigated algorithms. Notably, the LR model provides odds ratio estimates for risk factors only under conditions with numbers of variables less than 20 [39]. A complex dataset with many predictors make LR model difficult to specify all possible interactions [40]. In contrast, the computational power of the ANN is derived from the distributed nature of its connections. As such, the ANN can successfully manages complex datasets, even when the sample size is small or the ratio between variables and records is unbalanced [41], making it a natural modeling tool to examine survival in diverse populations [42]. In this study, we had included more variables such as preexisting comorbidities, helmet status, and laboratory data to improve prediction performance. Furthermore, the lack of specific mortality-related information in the trauma registry, including imaging characteristics of computed tomography scans [43], pupillary reactivity [7], and the existence of conscious deterioration, may have rendered the prediction model a space for improvement.

One criticism of the ANN is that it is difficult to assess the relative contribution of each variable to the final prediction put forth by the model [24]. Additionally, the ANN does not provide detailed information, such as the hazard ratio, which generally indicates the direction and magnitude of influence each variable has on the outcome [44]. Some other limitations included: First, patients declared dead on arrival at the hospital or at the scene of the accident were not recorded in the registered database [45, 46] and may have resulted in potential sample bias. The unknown status of inter-facility transfer or resuscitation for prehospital cardiac arrest may lead to a bias in the outcome measurement. Further, because the registered trauma data only had the in-hospital mortality but there were no information regarding the mortalities at 30 days, 3 months, or half a year data, there may exist some selection bias in the outcome measurement. Second, the imputation of physiological and laboratory data collected from the time of emergency department arrival does not reflect changes in hemodynamics and metabolic variables of patients who were under possible resuscitation. Third, this study was unable to assess the effects of any one particular treatment intervention, especially brain surgery. As such, we relied on the assumption that the assessment and management of patients—especially regarding operation quality—was uniform across the included population. Finally, the study sample was limited to a single urban trauma center in southern Taiwan, which may not be representative of other populations. However, because ANN generally could handle complex data which have interactions better than LR. Since ANN could do a better performance in mortality prediction than LR in this study according to the data from a single center, then we expect that ANN may still performed better than LR in dealing with the data from multicentric or multinational source. However, such opinion requires a further validation in the future study.

## Conclusion

We demonstrated that the ANN model provided better performance in predicting the mortality of patients with isolated moderate and severe TBI. The results of studies published so far are encouraging and may provide the first steps towards the development of a prediction model that can be integrated into trauma care systems to identify patients at high risk for mortality.

## Supporting information

S1 Fig Patient injury characteristics, as well as physiological and laboratory data collected on patient arrival to the emergency department. (TIF)

S2 Fig The independent risk predictors factors in logistic regression model. (TIFF)

S1 Table Statistical *p*-value among AUC comparisons between LR, SVM, DT, NB, and ANN models in the training set. (DOCX)
