# Supplementary figures and images for: Mortality prediction in patients with isolated moderate and severe traumatic brain injury using machine learning models
Source: PLoS One. 2018 Nov 9;13(11):e0207192. doi: 10.1371/journal.pone.0207192 (PMC6226171; doi:10.1371/journal.pone.0207192)

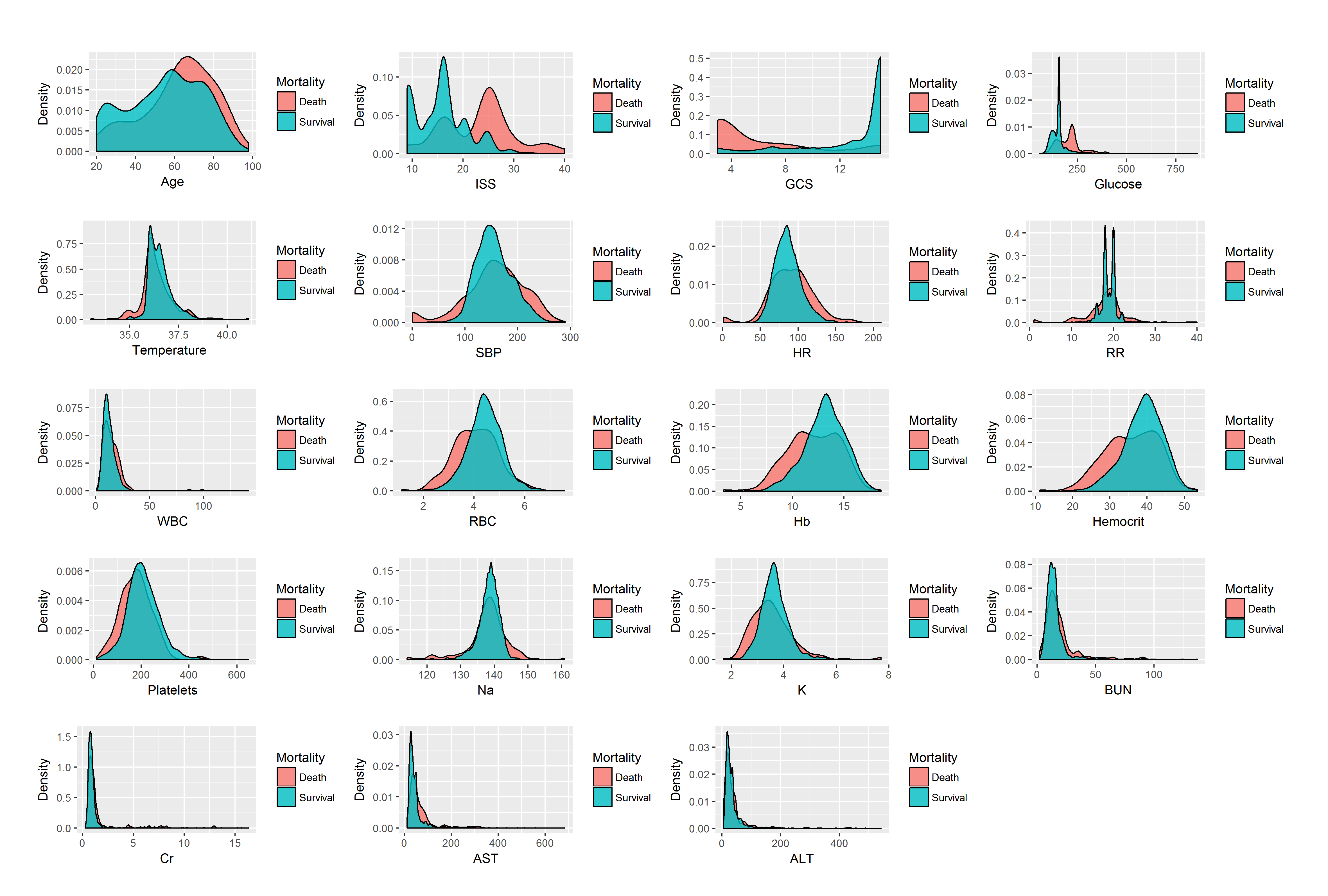

Supplement: S1 Fig — (TIF) [file pone.0207192.s001.tif]

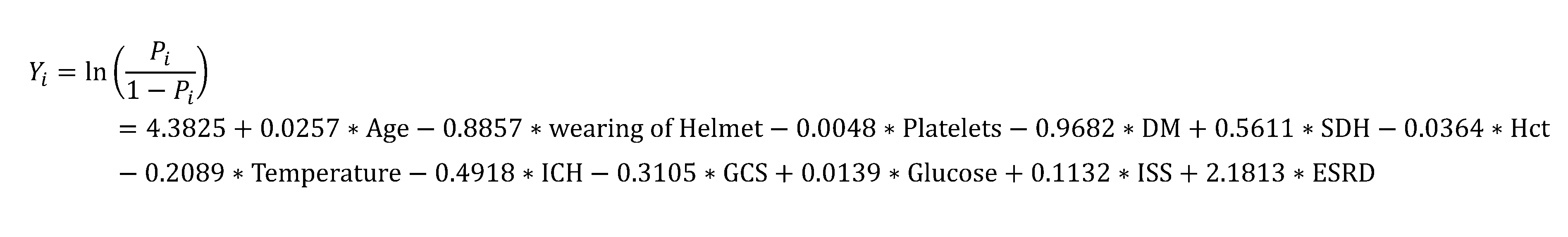

Supplement: S2 Fig — (TIFF) [file pone.0207192.s002.tiff]
