# Supplementary material for: Mortality prediction in patients with isolated moderate and severe traumatic brain injury using machine learning models
Source: PLoS One. 2018 Nov 9;13(11):e0207192. doi: 10.1371/journal.pone.0207192 (PMC6226171; doi:10.1371/journal.pone.0207192)
Supplement: S1 Table — (DOCX) [file pone.0207192.s003.docx]

Supplemental Digital Content - Table 1. Statistical *p*-value among under the curve (AUC) comparisons different kinds of machine learning models in the training set.

|  | LR | SVM | DT | Bayes |
| --- | --- | --- | --- | --- |
| LR | - | - | - | - |
| SVM | 0.1432 | - | - | - |
| DT | 0.0000 | 0.0000 | - | - |
| Bayes | 0.0002 | 0.0024 | 0.0254 | - |
| ANN | 0.0010 | 0.0001 | 0.0000 | 0.0000 |

LR, logistic regression; SVM, support vector machine; DT, decision trees; NB, Naive Bayes; and ANN, artificial neural networks.
